# Illinois

**Published:** 1896-08

**Authors:** 


*Illinois.* Forty of the herd of cows at the Kankakee Asylum were recently ordered destroyed on account of being tuberculous by Veterinary Trumbower

